# Non-bronchial systemic arteries in massive hemoptysis secondary to pulmonary tuberculosis: risk factors and outcomes of embolization materials. A retrospective observational study

**DOI:** 10.3389/fmed.2026.1877970

**Published:** 2026-08-06

**Authors:** Jingqiang Wu, Deqing Liu, Muhammad Tahir Khan, Peng Zhong, Yili Wang, Yongyong Le, Hongzhi Yang, Hui Zhang

**Affiliations:** 1 Institute of Tuberculosis, Guangzhou Medical University, Guangzhou, China; 2 Department of Radiology, Guangzhou Chest Hospital, Guangzhou, China; 3 Department of Tuberculosis, Guangzhou Chest Hospital, Guangzhou, China; 4 International Center for Interdisciplinary Research in Sciences (ICIRS), The University of Lahore, Lahore, Pakistan

**Keywords:** bronchial artery, embolization, massive hemoptysis, non-bronchial systemic artery, pulmonary tuberculosis

## Abstract

**Objectives:**

To investigate the distribution characteristics, independent risk factors of non-bronchial systemic arteries (NBSA) in patients with pulmonary tuberculosis.

**Methods:**

A retrospective analysis was performed using clinical data of 91 patients (75 males and 16 females) with pulmonary tuberculosis complicated by massive hemoptysis who underwent bronchial artery embolization (BAE). Patients were divided into the NBSA group (65 cases) and the non-NBSA group (26 cases). The NBSA group was further divided into the polyvinyl alcohol (PVA) particle group (45 cases) and the microsphere group (20 cases) based on the embolization material used. Univariate and multivariate COX regression analyses were used to identify independent risk factors for NBSA.

**Results:**

A total of 130 hemorrhagic lung lobes were involved in 91 patients (bilateral upper lobes accounting for 85.4%), with 281 responsible blood vessels, including 131 NBSAs. The main NBSAs were the posterior intercostal artery (41.98%), internal thoracic artery (35.11%), and lateral thoracic artery (9.16%). *Aspergillus* infection (OR = 87.909, *P* = 0.006) and pleural thickness ≥ 2 mm (OR = 8.532, *P* = 0.048) were independent risk factors for NBSA occurrence. There were no statistically significant differences in hemoptysis recurrence rate at 3 months and 1 year after surgery, median hemoptysis-free survival time, or incidence of adverse reactions between the PVA group and the microsphere group (all *P* > 0.05). The technical success rate of entire group was 100%, and the clinical effectiveness rate was 94.5%.

**Conclusion:**

Bronchial artery embolization demonstrated high technical success and clinical effectiveness in managing massive hemoptysis in pulmonary tuberculosis, with non-bronchial systemic arteries playing a significant role.

## 1 Background

Hemoptysis is a common complication of pulmonary tuberculosis, affecting approximately one-third of patients with the disease (1). Recurrent or massive hemoptysis is associated with a mortality rate as high as 75% (1, 2). Bronchial artery embolization (BAE) is regarded as the first-line interventional therapy for hemoptysis, as approximately 90% of the culprit vessels responsible for bleeding arise from the bronchial arterial (BA) circulation (3, 4). However, pulmonary tuberculosis lesions frequently involve the pleura, inducing pathological changes such as pleural thickening and adhesion. In such cases, systemic vessels other than the BA form neovascular anastomoses and thus emerge as crucial culprit vessels for hemoptysis; these vessels are collectively defined as Non-bronchial Systemic Arteries (NBSA) (5).

When NBSAs participate in the blood supply of the bleeding foci, simple BA embolization often yields suboptimal therapeutic outcomes, leading to hemoptysis recurrence and posing a major clinical challenge in clinical practice. Existing studies on NBSA embolization for hemoptysis have mostly focused on patients with bronchiectasis complicated by infection (6). In contrast, comprehensive large-sample investigations into the distribution characteristics, risk factors, and embolization efficacy of NBSAs in pulmonary tuberculosis complicated by massive hemoptysis remain scarce (7–9).

Pulmonary tuberculosis-related hemoptysis features a high incidence and is frequently complicated by concurrent infections with *Aspergillus* and non-tuberculous mycobacteria (NTM), which in turn induce pleural lesions. Additionally, post-tuberculous lung disease further leads to severe complications, including lung destruction and bronchiectasis. Under the influence of these complex pathological mechanisms, the distribution of NBSA culprit vessels is often more intricate in patients with tuberculosis-related hemoptysis.

We aimed to elucidate the distribution patterns and associated influencing factors of NBSAs, which hold great clinical significance for optimizing BAE therapeutic strategies, improving hemostatic success rates, and reducing the recurrence rate of hemoptysis. For this purpose, we performed a retrospective analysis of clinical data from patients with pulmonary tuberculosis complicated by hemoptysis, with a primary focus on investigating the distribution characteristics of pathological NBSAs and the efficacy of interventional embolization therapy, aiming to provide evidence-based references for clinical practice.

## 2 Materials and methods

### 2.1 Study design and patient selection

Ethical approval was obtained and all procedures were performed in compliance with relevant laws and institutional guidelines and have been approved by the institutional committee (2023) No. 37. The privacy rights of human subjects have been observed. A retrospective analysis was conducted on patients with hemoptysis who underwent bronchial artery embolization (with or without non-bronchial systemic artery embolization). The inclusion criteria were as follows: (1) Clinically diagnosed or etiologically confirmed pulmonary tuberculosis; (2) Patients with massive hemoptysis who underwent BAE (with or without NBSA embolization), including those with a single hemoptysis volume > 100 ml, >500 ml/24 h, or <100 ml but at risk of asphyxiation.

The exclusion criteria were as follows: (1) Incomplete clinical data or loss to follow-up; (2) Age < 18 years; (3) Complicated with lung cancer, and the bleeding was clinically considered to be caused by the tumor. All laboratory examinations and CT scan data were collected within 1 week of the start of BAE treatment.

### 2.2 Surgical methods: BAE and NBSA embolization

Systemic artery embolization, including BA and NBSA embolization, was performed under standard medical management. All operations were performed or supervised by experienced interventional radiologists. Percutaneous catheterization via the femoral artery was performed under local anaesthesia for arteriography. A 5F angiographic catheter was used for selective intubation and angiography of BA or NBSA. Abnormal arteries were identified by angiographic features and embolized as follows: (A) Abnormally dilated and tortuous blood vessels with rich pulmonary parenchymal staining; (B) Presence of bronchopulmonary shunt or contrast extravasation; (C) Aneurysm formation. Our embolization strategies were: (i) Bronchial artery embolization: After identifying the pathological blood vessels by angiography, a 2.7-F microcatheter (Terumo) was introduced. 300–500 μm polyvinyl alcohol (PVA) particles were mixed with 20 ml of contrast medium in a 20 ml reservoir syringe and injected slowly. The injection of PVA particles was stopped when the anterograde flow of the contrast embolization fluid slowed down. In some cases, after PVA embolization, 350–560 μm gelatin sponge particles were used for auxiliary embolization of proximal blood vessels. (ii) NBSA embolization: The embolization method for NBSA in some cases was the same as that for the bronchial artery. In other cases, embolization microspheres were used for NBSA embolization, which was due to the limited choice of materials at that time and the experience of the operating physicians. The method was to mix 300–500 μm embolization microspheres with 20 ml of contrast medium in a 20 ml reservoir syringe and inject slowly. The injection was stopped when the anterograde flow of the contrast embolization fluid slowed down.

### 2.3 Hemorrhagic lung lobes and responsible blood vessels

Preoperative CT images and intraoperative DSA angiographic images were analyzed by two radiologists specialized in vascular intervention as follows: Definition of hemorrhagic lung lobes: (A) Presence of alveolar hemorrhage: ground-glass opacity or consolidation; or visible blood clot obstruction in the bronchi; (B) Abnormal pulmonary parenchymal lesions complicated with bronchiectasis, cavitation, or lung destruction. Definition of responsible blood vessels: (A) All abnormal bronchial arteries identified by DSA angiography; (B) NBSA closely adjacent to the hemorrhagic lung lobe, with abnormal manifestations such as thickening, tortuosity, and bronchopulmonary shunt on DSA angiography.

### 2.4 Safety and efficacy evaluation

All patients with hemoptysis were followed up regularly after BAE, and adverse reactions within 1 week after surgery were recorded. The recording and evaluation of adverse events (AE) were performed in accordance with the Common Terminology Criteria for Adverse Events (CTCAE) Version 5.0.

Efficacy evaluation criteria: Technical success was defined as complete embolization of all attempted bleeding responsible blood vessels. Clinical effectiveness was defined as complete relief of hemoptysis symptoms or a significant reduction in hemoptysis volume (>50%) within 30 days after interventional embolization, without the need for further clinical intervention. Recurrent hemoptysis refers to repeated hemoptysis requiring clinical intervention (hemostatic drugs, re-embolization, hospitalization, etc.) after embolization. Recurrence within 30 days postoperatively is early recurrence, and beyond 30 days is late recurrence. Mild blood-tinged sputum without intervention is not counted as recurrence.

### 2.5 Follow-up

All patients received standard anti-tuberculosis treatment. Follow-up started after interventional embolization, with a uniform cutoff date of 1 September 2025. Follow-up was terminated immediately if hemoptysis recurred. Patients without recurrence were followed up every 3 months, including history taking, physical examination, laboratory tests and chest imaging. Follow-up duration was calculated to the date of the last visit; for those remaining recurrence-free by 1 September 2025, this date served as the follow-up endpoint.

### 2.6 Statistical methods

Statistical analysis was performed using SPSS 25.0 software, with α = 0.05 as the test level for intergroup comparisons. The normality of the measurement data was assessed using the Shapiro-Wilk test. Measurement data conforming to normal distribution were described as mean ± standard deviation (x ± s), while those not conforming to normal distribution were expressed as P50 (P25, P75). Categorical data were expressed as the number of cases and percentage (%). For intergroup comparisons of measurement data, a two-independent-samples *t*-test was used; if the conditions were not met, a Mann-Whitney U non-parametric rank-sum test was used. For intergroup comparison of categorical data, the chi-square test or Fisher’s exact test was used. Univariate analysis was performed to identify factors associated with NBSA occurrence. Factors with *P* < 0.05 in the univariate analysis were included in the multivariate analysis, and Cox regression was used to identify independent risk factors for NBSA. The Kaplan-Meier method was used to estimate survival curves, and the median time to recurrent hemoptysis was calculated using the Kaplan-Meier method. The difference was tested by the Log-rank test.

## 3 Results

### 3.1 Study population

During the study period, 375 patients with massive hemoptysis underwent BAE, of whom 91 met the inclusion and exclusion criteria. There were 75 males and 16 females, aged 18–84 years, with an average of 51.264 ± 15.176 years. The primary diseases of the patients were: pulmonary tuberculosis in 91 cases (100%); complicated with non-tuberculous mycobacterial lung disease (NTM lung disease) in 7 cases, bronchiectasis in 47 cases; complicated with *Aspergillus* in 39 cases; drug-resistant pulmonary tuberculosis in 2 cases, and pneumoconiosis in 5 cases (Table 1). Based on the presence or absence of NBSA on DSA angiography, patients were divided into two groups: 65 cases in the NBSA group and 26 in the non-NBSA group. According to the embolization material, the NBSA group was further divided into two subgroups: 45 cases in the PVA embolization group and 20 cases in the microsphere embolization group (Figurea 1). The baseline comparison of clinical characteristics between the two subgroups is shown in Table 2. There were no statistically significant differences in all clinical characteristics between the PVA group and the microsphere group (all *P* > 0.05).

**TABLE 1:** Etiological statistics *n* (%).

Etiology	Number (*n*/%)
Pulmonary tuberculosis	91 (100)
Complications	
Bronchiectasis	47 (51.6)
NTM lung disease	7 (7.7)
*Aspergillus* infection	39 (42.9)
Drug-resistant pulmonary tuberculosis	2 (2.2)
Pneumoconiosis	5 (5.5)

**FIGURE 1:**
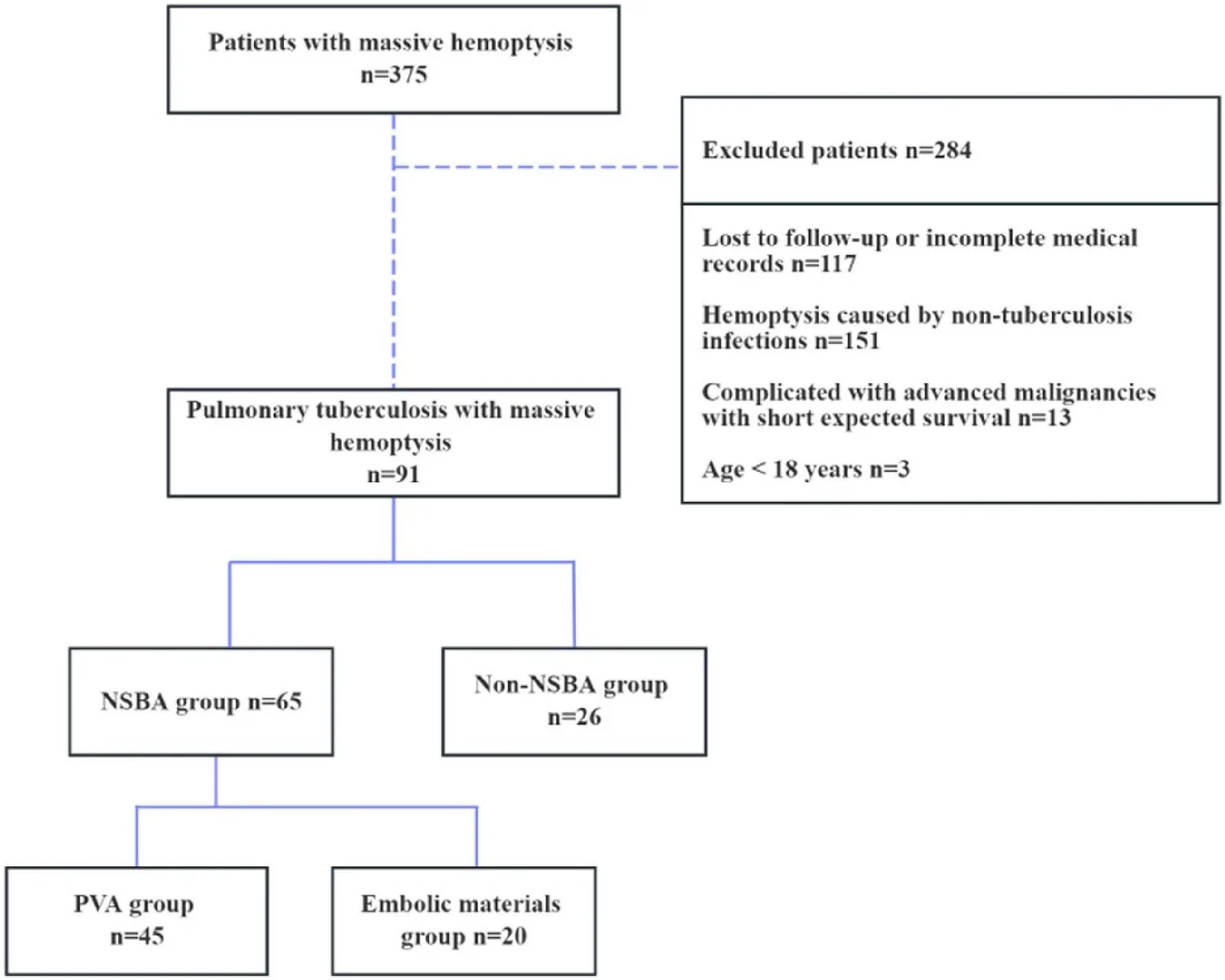
Flowchart methodology.

**TABLE 2:** Baseline comparison of clinical characteristics between the polyvinyl alcohol (PVA) group and the microsphere group *n* (%), x ± s, M (P25, P75).

Index	Characteristics	PVA group (A)	Microsphere group (B)	χ^2^/*t*/*Z*/Fisher	*P*
*n*		45	20	Value	–
Gender				0.000*	1.000
	Male	39 (86.7)	18 (90.0)	–	–
	Female	6 (13.3)	2 (10.0)	–	–
Age		52.111 ± 15.618	46.300 ± 15.190	1.396^#^	0.168
Drug-resistant pulmonary TB				–♣	1.000
	Yes	44 (97.8)	20 (100.0)		
	No	1 (2.2)	0 (0.0)		
*Aspergillus* infection				3.374*	0.066
	Yes	16 (35.6)	12 (60.0)	–	–
	No	29 (64.4)	8 (40.0)	–	–
NTM lung disease				–♣	1.000
	Yes	2 (4.4)	1 (5.0)	–	–
	No	43 (95.6)	19 (95.0)	–	–
Bronchiectasis				2.708*	0.100
	Yes	15 (33.3)	11 (55.0)	–	–
	No	30 (66.7)	9 (45.0)	–	–
Pneumoconiosis				–♣	0.547
	Yes	42 (93.3)	20 (100.0)	–	–
	No	3 (6.7)	0 (0.0)	–	–
Duration of illness (years)		3.000 (1.000, 8.500)	3.500 (0.550, 9.000)	−0.493^Δ^	0.622
Hemoptysis volume (mL)		100.000 (50.000, 200.000)	100.000 (35.000, 300.000)	−0.108^Δ^	0.914

*Chi-square test. #*t*-test. ^Δ^*Z*-test. ♣Fisher’s exact test.

### 3.2 Distribution of hemorrhagic lung lobes and responsible blood vessels

Among the 91 patients with pulmonary tuberculosis complicated by massive hemoptysis who underwent BAE, the technical success rate was 100%; 5 cases (5.5%) had recurrent hemoptysis in the short term, 86 cases had no recurrent hemoptysis, and the clinical effective rate was 94.5%. A total of 130 hemorrhagic lung lobes were involved, including 59 in the right upper lobe, six in the right middle lobe, five in the right lower lobe, 52 in the left upper lobe, and 8 in the left lower lobe. A total of 281 responsible blood vessels were involved, including 150 BAs and five ectopic bronchial arteries. In addition, there were 131 NBSAs, including 55 posterior intercostal arteries, one proper oesophagal artery, 46 internal thoracic arteries, eight thyrocervical trunks, five costocervical trunks, 12 lateral thoracic arteries, one superior thoracic artery, one subscapular artery, and two inferior phrenic arteries. The top three non-bronchial systemic hemorrhagic arteries were the posterior intercostal artery, the internal thoracic artery, and the lateral thoracic artery, accounting for 41.98%, 35.11%, and 9.16%, respectively. The hemorrhagic lung lobes were mainly the bilateral upper lobes, and the NBSAs were dominated by the posterior intercostal and internal thoracic arteries (Table 3).

**TABLE 3:** Distribution of hemorrhagic lung lobes and responsible blood vessels.

Arteries	Right upper lobe (59)	Right middle lobe (6)	Right lower lobe (5)	Left upper lobe (52)	Left lower lobe (8)
Bronchial artery/ectopic bronchial artery	73/5	7/0	7/0	50/0	8/0
Posterior intercostal artery/esophageal artery/inferior phrenic artery	27/0/0	–	–	28/1/0	0/0/2
Thyrocervical trunk/costocervical trunk/internal thoracic artery	2/3/20	0/0/1	–	6/2/24	0/0/1
Superior thoracic artery/lateral thoracic artery/subscapular artery	0/8/1	–	–	1/4/0	–

### 3.3 NBSA occurrence

The 91 patients were divided into the NBSA and non-NBSA groups. Univariate analysis was used to examine the relationship between the two groups and eight observation indicators, including *Aspergillus* infection, bronchiectasis, course of disease, lesion nature, presence or absence of a cavity, cavity size, pleural thickness, and relationship between the lesion and the pleura. The data showed that the NBSA group had a higher incidence of *Aspergillus* infection and bronchiectasis, as well as longer disease duration, higher lesion necrosis rate, higher cavitation rate, larger cavities, thicker pleura, and a higher degree of adhesion between the lesion and the pleura. There were no statistically significant differences in other indicators between the two groups (all *P* > 0.05). The above eight indicators were included in multivariate analysis, and the results suggested that *Aspergillus* infection and pleural thickness ≥ 2 mm were independent risk factors for NBSA occurrence (Table 4).

**TABLE 4:** Univariate and multivariate analyses of non-bronchial systemic arteries (NBSA) occurrence *n* (%), x ± s, M (P25, P75).

Index	Non-NBSA group (*n* = 26)	NBSA group (*n* = 65)	Univariate analysis		Multivariate analysis		
			χ^2^/*t*/*Z*/Fisher	*P*	OR	95% CI	*P*
Gender	–	–	3.187*	0.074	–	–	–
Male	18 (69.2)	57 (87.7)	–	–	–	–	–
Female	8 (30.8)	8 (12.3)	–	–	–	–	–
Age	49.439 ± 16.657	52.760 ± 13.837	−1.039#	0.302	–	–	–
*Aspergillus* infection	–	–	22.620*	**0.000**	87.909	3.6–2124.8	**0.006**
No	25 (96.2)	27 (41.5)	–	–	–	–	–
Yes	1 (3.8)	38 (58.5)	–	–	–	–	–
Bronchiectasis			6.354*	**0.012**	0.166	0.015–1.79	0.139
No	18 (69.2)	26 (40.0)	–	–	–	–	–
Yes	8 (30.8)	39 (60.0)	–	–	–	–	–
Duration of illness (years)	2.0 (0.250, 5.0)	4.00 (2.000, 10.0)	−2.740Δ	**0.006**	0.820	0.632–1.065	0.137
Hemoptysis volume (mL)	100.0 (40.0, 200.0)	100.0 (50.0, 200.0)	−0.890Δ	0.374	–	–	–
Lesion involvement range	–	–	1.695*	0.193	–	–	–
Limited to 1 lobe	7 (26.9)	27 (41.5)	–	–	–	–	–
2 or more lobes	19 (73.1)	38 (58.5)	–	–	–	–	–
Lesion nature	–	–	6.076*	**0.048**	4.267	0.4–45.2	0.229
Necrosis	7 (26.9)	36 (55.4)	–	–	–	–	–
Exudation	8 (30.8)	13 (20.0)	–	–	–	–	–
Fibroproliferation	11 (42.3)	16 (24.6)	–	–	–	–	–
Lung destruction	–	–	3.539*	0.060	–	–	–
No	26 (100.0)	54 (83.1)	–	–	–	–	–
Yes	0 (0.0)	11 (16.9)	–	–	–	–	–
Cavity	–	–	6.376*	**0.012**	0.067	0.004 to −1.0	0.050
No	12 (46.2)	13 (20.0)	–	–	–	–	–
Yes	14 (53.8)	52 (80.0)	–	–	–	–	–
Cavity size (cm)	1.000 (0.000, 3.350)	3.750 (1.875, 5.250)	−3.914Δ	**0.000**	1.916	0.996–3.689	0.052
Pleural thickness	–	–	17.102*	**0.000**	8.532	1.015–71.7	**0.048**
<2 mm	19 (73.1)	17 (26.2)	–	–	–	–	–
≥2 mm	7 (26.9)	48 (73.8)	–	–	–	–	–
Relationship between lesion and pleura	–	–	22.750*	**0.000**	2.436	0.416–14.2	0.323
Mild adhesion or none	20 (76.9)	15 (23.1)	–	–	–	–	–
Tight adhesion	6 (23.1)	50 (76.9)	–	–	–	–	–
Pleural effusion	–	–	0.190*	0.663			
No	23 (88.5)	61 (93.8)	–	–	–	–	–
Yes	3 (11.5)	4 (6.2)	–	–	–	–	–

*Chi-square test. #*t*-test. Bold: Statistically significant.

### 3.4 Comparison of embolization efficacy and safety between the PVA group and the microsphere group

In the PVA group, 1 case had recurrent hemoptysis 3 months after surgery, and 8 cases had recurrent hemoptysis 1 year after surgery. In the microsphere embolization group, 2 cases had recurrent hemoptysis 3 months after surgery, and 4 cases had recurrent hemoptysis 1 year after surgery. There were no statistically significant differences in the hemoptysis recurrence rate between the two groups at 3 months and 1 year after surgery (all *P* > 0.05) (Table 5).

**TABLE 5:** Therapeutic effects of the two groups at 3 months and 1 year after surgery *n* (%).

Time	*n*	Postoperative 3 months		Postoperative 1 year	
group		Recurrent hemoptysis	No recurrent hemoptysis	Recurrent hemoptysis	No recurrent hemoptysis
PVA group	45	1 (2.2)	44 (97.8)	8 (18.2)	36 (81.8)
Microsphere group	20	2 (10.0)	18 (90.0)	4 (22.2)	14 (77.8)
χ^2^/Fisher	–	–♣		0.000*	
*P*	–	0.222		0.991	

*Chi-square test. ♣Fisher’s exact test.

In the PVA group, 17 cases developed chest and back pain after surgery, and 7 cases developed fever, all of which were grade 2 adverse reactions, with no grade 3 or above complications. In the microsphere embolization group, 5 cases developed chest and back pain after surgery, and 3 cases developed postoperative fever, all of which were grade 2 adverse reactions; 1 case developed aortic dissection during surgery requiring prolonged hospital stay, which was a grade 3 adverse event. There were no statistically significant differences in the incidence of chest and back pain, fever, or severe complications between the two groups (all *P* > 0.05) (Table 6).

**TABLE 6:** Safety comparison between the two groups [*n* (%)].

Group	*n*	Chest and back pain	Fever	Severe complications
PVA group	45	17 (37.8)	7 (15.6)	0
Microsphere group	20	5 (25.0)	3 (15.0)	1 (5.0)
χ^2^/Fisher	–	1.010*	0.000*	–*
*P*	–	0.315	1.000	0.308

*Chi-square test.

### 3.5 Kaplan-Meier curve of hemoptysis-free survival time

**FIGURE 2:**
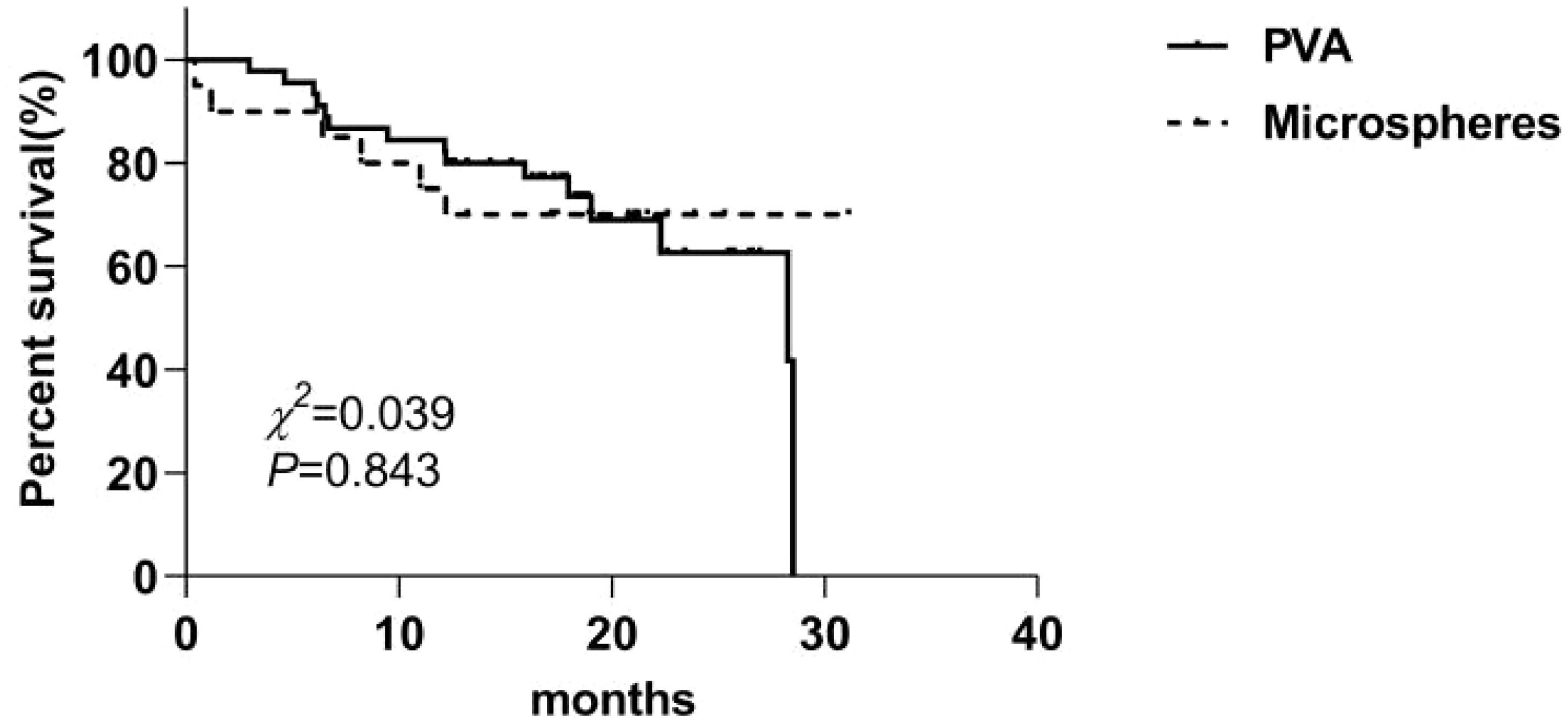
Kaplan-Meier survival analysis comparing polyvinyl alcohol (PVA) and microsphere treatment groups.

A total of 91 patients were included in this study, among which 65 were followed up until 1 September 2025. The follow-up duration ranged from 0.4 to 31.13 months, with a median follow-up time of 17.63 months. Recurrent hemoptysis did not occur in 44 cases (30 in the PVA group and 14 in the microsphere group). The hemoptysis-free survival time in the PVA group was 22.692 months (98% CI 19.949–25.436), and that in the microsphere group was 23.765 months (98% CI 18.716–28.814). There was no statistically significant difference in the hemoptysis-free survival time between the two groups (*P* = 0.843, Figure 2).

Figure 2 Kaplan-Meier curve of hemoptysis-free survival time between the PVA group and the microsphere group.

Survival over a 40-month follow-up period for patients treated with polyvinyl alcohol (PVA) versus microspheres.

## 4 Discussion

Pulmonary tuberculosis is the leading cause of hemoptysis in developing countries, accounting for approximately 65% (7, 8). Even after standardized treatment, the structural lung injury caused by it is prone to recurrent hemoptysis, which is life-threatening in severe cases. The blood supply for hemoptysis is mainly from BA. Still, the role of NBSA in the management of massive hemoptysis due to pulmonary tuberculosis has received increasing attention (9). Nishihara et al. (10) confirmed that NBSAs in patients with pulmonary tuberculosis is a key factor in massive hemoptysis in pulmonary tuberculosis, consistent with the core logic of this study, which focuses on NBSAs.

Through univariate and multivariate analyses, this study identified *Aspergillus* infection and pleural thickness ≥ 2 mm as independent risk factors for NBSA occurrence in patients with pulmonary tuberculosis (OR 87.909 and 8.532, *P* < 0.05), consistent with a previous study (11, 12). From a pathological perspective, chronic inflammation associated with pulmonary tuberculosis can lead to thickening and adhesion of the visceral and parietal pleura. Hypoxia in the alveolar interstitium and proliferation of granulation tissue can induce the release of angiogenic factors, promoting the formation of new blood vessels and their anastomosis with adjacent NBSAs, resulting in bronchopulmonary shunts (BPS), which further increases the risk of hemoptysis (13, 14). In *Aspergillus* infection, the proteases, phospholipases, and other virulence factors secreted by its hyphae can damage the connections between vascular endothelial cells, triggering a strong inflammatory response, leading to congestion, lung tissue necrosis, damage to vascular wall structure, and even the formation of pseudoaneurysms. At the same time, hypoxia and structural remodeling can further open the pulmonary artery-bronchial artery anastomotic plexus, accelerating the formation of collateral circulation (15, 16). These two factors together constitute the core pathological basis for NBSA occurrence.

Clarifying the distribution regularity of NBSA is crucial for improving the accuracy of embolization treatment. This study showed that the hemorrhagic lung lobes in massive hemoptysis due to pulmonary tuberculosis were mainly the bilateral upper lobes (59 in the right upper lobe and 52 in the left upper lobe), which is consistent with the pathological characteristic that pulmonary tuberculosis lesions are more likely to occur in the apical posterior segment of the upper lobe and the dorsal segment of the lower lobe. Among the 131 NBSAs, the top three responsible blood vessels were the posterior intercostal artery (41.98%), internal thoracic artery (35.11%), and lateral thoracic artery (9.16%) (17, 18). This distribution characteristic suggests that, in clinical interventional treatment, for patients with bilateral upper lobe bleeding, it is necessary to focus on exploring the posterior intercostal and internal thoracic arteries to avoid recurrent hemoptysis due to missed NBSA. Keshmiri et al. (19) proposed that 92% of hemoptysis is from the BA system, 3% from NBSA, and 5% from both BA and NBSA. In this study, in addition to BA, NBSA was involved in the blood supply in 46.6% (131/281) of the responsible blood vessels for hemoptysis. This may be related to the study population consisting solely of patients with pulmonary tuberculosis and a high proportion of complicated pleural lesions, further confirming the particularity of NBSA involvement in hemoptysis in patients with pulmonary tuberculosis (20).

The choice of embolization materials directly affects the therapeutic efficacy and safety. In some retrospective analysis studies (21, 22), the recurrence rate of hemoptysis after BAE using permanent embolization materials is significantly lower than that using short-acting embolization materials, and the efficacy of embolization with PVA or microspheres is comparable. However, there are few comparative studies on materials for NBSA embolization. In this study, patients who underwent NBSA embolization were divided into the PVA group (45 cases) and the microsphere group (20 cases). The results showed that there were no statistically significant differences in the hemoptysis recurrence rate at 3 months (2.2% vs 10.0%) and 1 year (18.2% vs 22.2%) after surgery, as well as the median hemoptysis-free survival time (22.692 vs 23.765 months) between the two groups (all *P* > 0.05). In terms of safety, there were no significant differences in the incidence of adverse reactions, including chest and back pain, fever, or severe complications, between the two groups (all *P* > 0.05). This result indicates that PVA and microspheres have comparable efficacy and safety in NBSA embolization, both of which are feasible clinical choices, consistent with the research conclusions of Bruce-Brand et al. (23, 24), providing a flexible basis for clinical material selection.

This study has certain limitations: First, it is a single-center retrospective study with a relatively small sample size of 91 cases, which may introduce selection bias; its conclusions need to be further verified by multi-centre, large-sample studies. Second, the median follow-up time was 17.63 months, which did not cover the 3–5-year long-term follow-up period, so the long-term stability of embolization materials and the risk of NBSA recanalization could not be fully evaluated.

## 5 Conclusion

This study systematically clarified the distribution characteristics and key risk factors of NBSA in patients with pulmonary tuberculosis complicated by massive hemoptysis. It confirmed the clinical application value of PVA and microspheres. In clinical practice, preoperative CT assessment of *Aspergillus* infection and pleural thickness (≥2 mm) can efficiently identify high-risk groups for NBSA. Intraoperative focus on exploring dominant responsible blood vessels, such as the posterior intercostal artery and internal thoracic artery, can improve the comprehensiveness and accuracy of embolization treatment and ultimately reduce the recurrence rate of hemoptysis. This study provides important evidence-based support for optimizing the interventional treatment strategy of massive hemoptysis due to pulmonary tuberculosis.

## Data Availability

The raw data supporting the conclusions of this article will be made available by the authors, without undue reservation.
